# The Utility of Nanocomposites in Fire Retardancy

**DOI:** 10.3390/ma3094580

**Published:** 2010-09-03

**Authors:** Linjiang Wang, Xuejun He, Charles A. Wilkie

**Affiliations:** 1 Key Laboratory of New Processing Technology for Nonferrous Metals and materials, Ministry of Education, Guilin University of Technology, Guilin 541004, China; E-Mail: guthxj@gmail.com; 2 Department of Chemistry and Fire Retardant Research Facility, Marquette University, Milwaukee, WI 53201, USA

**Keywords:** fire retardancy, nanocomposites, clays

## Abstract

Nanocomposites have been shown to significantly reduce the peak heat release rate, as measured by cone calorimetry, for many polymers but they typically have no effect on the oxygen index or the UL-94 classification. In this review, we will cover what is known about the processes by which nanocomposite formation may bring this about. Montmorillonite will be the focus in this paper but attention will also be devoted to other materials, including carbon nanotubes and layered double hydroxides. A second section will be devoted to combinations of nanocomposite formation with conventional (and unconventional) fire retardants. The paper will conclude with a section attempting to forecast the future.

## 1. Introduction

Nanocomposites have received much interest over the past decades due to their significant advantages over conventional composites, in which high loadings of additives are often required [1,2,3,4,5,6,7,8]. The nanometer-scale material which has been most investigated is layered clay, primarily including layered silicates (montmorillonite (MMT) is the most studied member of this family) [9,10,11]. Other nanometer dimension materials that have been studied include layered double hydroxides (LDHs) [12,13,14,15] and carbon nanotubes, either single-, double- or multi-wall [16,17,18,19,20,21,22,23,24,25].

Polymer-layered clay nanocomposites were reported in the patent literature as early as 1950 [26], while polyamide nanocomposites were reported in 1976 [27]. However, it was not until the Toyota research group began a detailed examination of polymer layered clay mineral composites that nanocomposites became more widely studied in academia [28]. Composites formed by the combination of polymer and additives have properties superior to those of the individual constituents, primarily because there is now a large interfacial region which dominates the properties of the system. The structures and properties of composite materials can be significantly influenced by morphologies, interfacial properties and dispersion of the additives in the polymer matrix.

Polymer-MMT nanocomposites are the most common class of nanocomposites and the one which has been most investigated [29,30,31,32]. Because of the dispersion at the nanometer level, polymer-clay nanocomposites exhibit superior properties in comparison with pure polymer or conventional composites; this includes properties such as light weight [33,34], high modulus, enhanced physical-mechanical properties [35,36,37], barrier properties [38,39], increased solvent resistance [40,41], improved thermal stability and flame retardancy [42,43,44]. Another impressive feature of nanocomposites and nano-filled composites is the concurrent improvement of multiple properties, in addition to the introduction of new functionalities [33,34]. The mechanical properties of nanocomposites are superior to the fiber-reinforced polymers because enhancement from the inorganic layers occurs in three dimensions rather than only in the dimension of the fiber [32]. Improvements in thermal stability of polymer-clay nanocomposites is due to the nano-sized layers restricting the polymer molecular chain motion [45].

The outstanding properties of polymer-clay nanocomposites are achieved at a much lower volume fraction, compared with conventional composites. For instance, when the clay content is as low as 1% in a polystyrene/layered silicate nanocomposite, the initial decomposition temperature increases by 40 °C and the peak heat release rate decreases by 40% compared with virgin PS [2]. Polymer-clay nanocomposites can be processed using common techniques, such as extrusion and casting, which are superior to the cumbersome techniques used for the conventional composites. In addition, polymer-clay nanocomposites could be used to manufacture films, fibers and monoliths. Hence, polymer-clay nanocomposites have important potential commercial value [46]. In this review, we will focus on the formation and characterization of nanocomposites, fire retardancy and its mechanism and an evaluation of fire retardancy, mainly based on cone calorimetric analysis.

## 2. Nanocomposite Formation and Characterization

### 2.1. Formation of Nanocomposites

Not all polymer and inorganic additive combinations will form nanocomposites: the compatibility and interfacial properties between polymer matrix and inorganic additives significantly influence the essential characteristics of materials [46]. Generally, inorganic additives have poor compatibility with the polymer matrix, except for water soluble polymers. Therefore inorganic additives must be organically-modified, using organic surfactants, to improve compatibility. The organic surfactants in the organically-modified additives play the important role of lowering the surface energy of the inorganic host, improving the wetting characteristics and miscibility with the polymer matrix [29,30,31,44,47,48].

Nanocomposites can be formed by the following four principal methods: (i) *in-situ* template synthesis, (ii) polymerization techniques, (iii) solvent based blending and (iv) melt blending [29,49,50].

#### 2.1.1. *In-situ* Template Synthesis (sol-gel technique)

This procedure is based on the synthesis of the inorganic host in the presence of a polymer that acts as a templating agent for the growing solid, in a way similar to alkylammonium ions or surfactants that are used in the formation of certain zeolites or mesoporous silica materials [49]. This procedure derives from synthetic inorganic hosts, such as layered double hydroxides (LDHs). In the case of clays, this route is relatively new and was applied for the first time by Carrado and coworkers [51] to obtain polymer–hectorite nanocomposites. The clay minerals are synthesized within the polymer matrix, using an aqueous solution (or gel) containing the polymer and the silicate building blocks. Magnesium hydroxide sol and lithium fluoride are utilized as precursors for the silica sol. During the preparation, the polymer aids the nucleation and growth of the inorganic host and is trapped within the layers as the inorganic host grows. Theoretically, this technique could promote the dispersion of the layers of clay in a one-step process. However, it presents serious disadvantages. For example, the temperature required for the synthesis of clay minerals is generally high, which could cause the decomposition of polymer and, with the growth of clay layers, aggregation will occur. Carrado synthesized hectorites from sols consisting of silica, magnesium hydroxide, lithium fluoride and polymers like poly(vinyl alcohol), polyaniline and polyacrylonitrile. Some silicate layers aggregated, but most of them remained uniformly distributed in the polymer matrix [49].

#### 2.1.2. Polymerization Techniques

*In-situ* polymerization is one of the most widely used techniques to prepare polymer-layered clay nanocomposites. During the synthesis, the clay is dispersed in the monomer and the polymerization of the monomer is carried out. Polymerization can also occur in solution; Wang reported a comparison of bulk, solution, emulsion and suspension polymerization for PMMA and PS nanocomposites. One is more likely to obtain exfoliation using bulk, emulsion or suspension polymerization than with solution polymerization [52]. Tasdelen *et al.* [53] reported that a wide variety of polymer/clay nanocomposites can be synthesized by *in situ* living and controlled/living polymerization methods. The silicate layers can be either delaminated first, followed by a polymerization step, or can be exfoliated during polymerization by triggering polymer chain growth within the clay galleries. An exfoliated Co-Al layered double hydroxide/polyamide 6 nanocomposite was prepared by *in-situ* polymerization [54]. Park *et al.* [55] successfully prepared polyimide/single-wall carbon nanotubes nanocomposites under sonication by *in-situ* polymerization and investigated the dispersion of carbon nanotubes in the polymer matrix.

#### 2.1.3. Solvent-Assisted Blending

The solvent based process involves mixing a preformed polymer solution with the layered clay [46]. The layered clay can be exfoliated into single layers due to the weak forces between the layers, using a solvent in which the polymer could be dissolved. When the layered clay has been expanded in the solvent, the polymer is added to the solution and then it intercalates between the clay layers. In the next step, the solvent is removed, either by vaporization or by precipitation of the polymer [49]. However, this is difficult to use in industry because large amounts of solvents are used during the synthesis, which is environmentally disadvantageous [49,56]. Acrylonitrile-butadiene-styrene/montmorillonite nanocomposites were prepared by Pourabas *et al.*, using solvent blending [57]. Qiu *et al.* reported the preparation of exfoliated polystyrene/Zn-Al layered double hydroxides nanocomposite by solution intercalation, using xylene as solvent [58].

#### 2.1.4. Melt Blending

Melt blending is the technique most utilized for the preparation of nanocomposites and it is suitable for large scale production in industry. This technique utilizes the existing conventional polymer processing equipment, such as extrusion, roll mixing, batch and static mixing, *etc.* [44]. The melt blending process involves mixing the layered clay with the polymer while heating the mixture above the softening point of the polymer. During the mixing process, the molecular chain of the polymer matrix diffuse from the bulk polymer and melt into the galleries between the clay layers [46]. Su *et al.* [59] prepared polymer nanocomposites of poly (methyl methacrylate), polypropylene and polyethylene by melt blending with polymerically-modified montmorillonite. Polyamide-12/tetrasilisic fluoromica and polyamide-12/quaternary tallow ammonium chloride modified fluoromica nanocomposites were prepared by melt compounding [60]. Wang *et al.* [61] reported the use of montmorillonite, layered double hydroxide and kaolinite to make (nano) composites with poly (methyl methacrylate) by melt blending. Zhang *et al.* utilized single wall carbon tubes and high density polyethylene to prepare nanocomposites [62].

### 2.2. Characterization of Nanocomposites

It is important that the nanocomposites are carefully investigated by various analytical methods to determine if a nanocomposite has been formed and how the clay layers are arranged. Many methods are used to characterize the nanocomposites, including X-ray diffraction (XRD), transmission electron microscopy (TEM), nuclear magnetic resonance (NMR) spectroscopy, atomic force microscopy (AFM), scanning electron microscopy (SEM), *etc.* The most frequently used technique for characterization of nanocomposites is the combination of XRD and TEM.

#### 2.2.1. X-ray Diffraction

It is well known that only materials ordered enough to diffract X-ray can be detected; disordered materials will show no pattern with the X-ray technique [57]. The layer spacing of clay in the polymer matrix can be calculated using Bragg’s law: sin θ = n λ/2d [46,63]. Generally, the formation of an intercalated nanocomposite results in an increase in basal spacing in the XRD pattern, while the formation of an exfoliated nanocomposite leads to the complete loss of registry between the layers and therefore no peak can be observed. In general, the appearance of a strong peak at lower values of 2θ is probably indicative of an intercalated structure but the presence of a broad peak at any 2θ leaves open the possibility of disorder; this disorder could be caused by exfoliation or it could be a simple composite which is disordered. XRD is insufficient to characterize the nanocomposites structure and additional analytical techniques must be utilized to confirm the morphology of a material and explain the meaning of the XRD signal [57].

#### 2.2.2. Transmission Electron Microscopy

Complementary to XRD, TEM is the most popularly employed technique to determine nanocomposite morphology [57]; using TEM one can image the nanocomposite structure. In general, one collects several images at high and low magnification and at several positions in the nanocomposite sample. Both a low magnification image, to show the global dispersion of the additives in the polymer, and a higher magnification image, to evaluate the registry of additives are needed [64].

#### 2.2.3. Nuclear Magnetic Resonance (NMR) Spectroscopy

The interpretation of TEM images tends to be very subjective; the person who has made the system almost always sees more exfoliation than others may see. The NMR technique offers an opportunity to quantify, in a way, the type of dispersion. The main objective in solid-state NMR measurement is to connect the measured longitudinal relaxation times, T1^H^s, of proton with the quality of clay dispersion; the extent and the homogeneity of the dispersion of the silicate layers within the polymer matrix are very important for determining physical properties [65,66,67,68,69,70].

For the layered silicates (such as montmorillonite), the octahedrally coordinated Al^3+^ in the basal layers is often replaced by Fe^3+^. The presence of Fe^3+^ in the montmorillonite structure facilitates the relaxation of nearby protons, which can provide information on the dispersion of the clay in the nanocomposites. The relaxation time depends on how close the proton is to a paramagnetic iron atom. Generally, the protons of the polymers will be closer to the iron in the clay in an exfoliated system and thus will have the smallest relaxation time. For the microcomposite, the protons will be farthest from the iron and show a larger relaxation time. This information can be correlated with TEM and XRD information and can also be used as a stand-alone technique to ascertain morphology. However, this technique has its limitations. The layered silicates are naturally-occurring compounds and so the amount and distribution of iron may vary from one lot to the next. When solid state NMR technique is utilized for the characterization of nanocomposites, the same batch of clay must be used [44].

#### 2.2.4. Other Characterization Techniques for Nanocomposites

Other characterization technique have also been utilized to characterize the structure and properties of polymer nanocomposites, such as atomic force microscopy [71,72,73], X-ray photoelectron spectroscopy [71,74], fluorescence [75,76] and rheology [77,78], *etc.*

### 2.3. Nanocomposite Description

**Figure 1:**
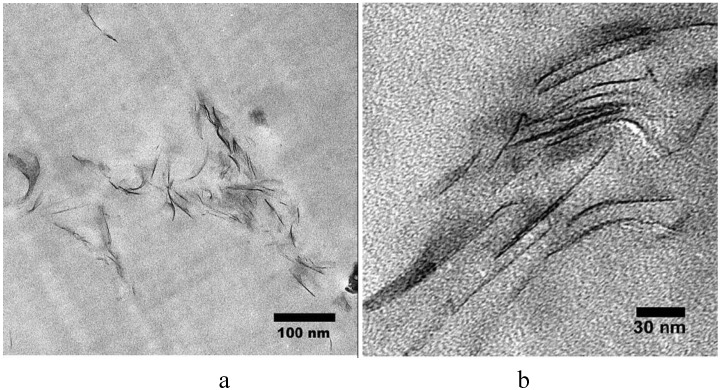
TEM micrographs of PS/MMT nanocomposites (a) at low magnification. (b)at higher magnification. Reproduced with permission from reference [82].

**Figure 2:**
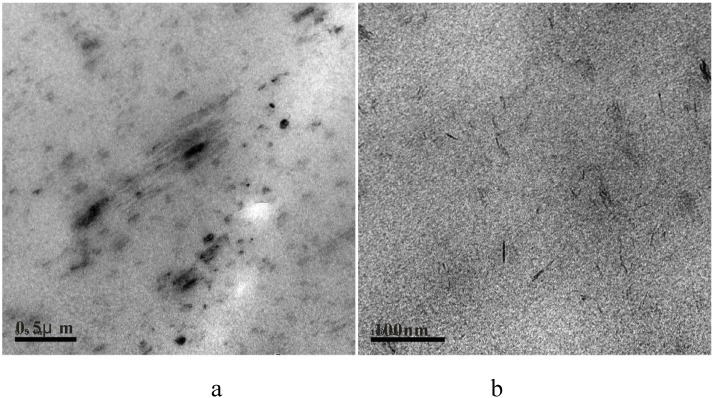
TEM micrographs showing exfoliated/intercalated/ immiscible LDH particles in PMMA (a) at low magnification. (b)at higher magnification. Reproduced with permission from reference [64].

Nanocomposites may be described as either immiscible, intercalated or exfoliated (also called delaminated); another possible description is an end tethered structure [49,56]. An immiscible nanocomposite is a conventional composite in which the clay is not separated into layers but rather only aggregates of clay are present. Intercalated structures are formed when a single (or more) extended polymer chain is intercalated between the layers of clay. The result is a well-ordered multilayer structure of alternating polymeric and inorganic layers, with a repeat distance between them; intercalation causes about 2-3nm separation between the platelets [28]. Exfoliated (delaminated) structures are formed when the clay layers are well separated from one another and individually dispersed within the continuous polymer matrix. In an intercalated structure, registry is maintained between the clay layers while registry is lost in an exfoliated structure. Because exfoliated nanocomposites have higher phase homogeneity than the intercalated counterpart, the exfoliated structure is more desirable in enhancing the properties of the nanocomposites. The exfoliated configuration is of particular interest because it maximizes the polymer-clay interactions making the entire surface of layers available for the polymer, which should lead to the most significant changes in mechanical and physical properties [56]. However, it is not easy to achieve complete exfoliation of clays and, indeed with few exceptions, the majority of the polymer nanocomposites reported in the literature were found to have intercalated nanostructures [79]. One may, in fact, make the case that it is rare to form only a single morphology and that most often one obtains a mixed morphology and mixture of all three possibilities is more common that one may expect, *i.e.*, mixed immiscible-intercalated-exfoliated morphology may be very common. TEM images for PS-MMT and PMMA-LDH are shown in Figure 1 and Figure 2; the aspect ratio (ratio of length to thickness) is significantly larger for MMT than for LDH, which has an effect on the mechanical properties.

Two types of end-tethered structures can be produced, one where the end of the polymer is attached to the outside of the silicate sheet and the other where the end of the polymer is attached to an exfoliated layer of the silicate. The second type is similar to a delaminated structure with polymer surrounding exfoliated layers of silicate [80,81].

## 3. Evaluation of Fire Retardancy

The main evaluation methods for the fire retardancy of nanocomposites include thermal stability, cone calorimetry, limiting oxygen index and the UL-94 protocol.

### 3.1. Thermal Stability

The thermal stability of polymeric materials is usually studied by thermogravimetric analysis (TGA). The weight loss due to the formation of volatile products after degradation is monitored as a function of temperature (and/or time). When heating occurs under an inert gas flow, a non-oxidative degradation occurs, while the use of air or oxygen allows oxidative degradation of the samples [49]. The data obtained from TGA include the onset temperature of the degradation, typically taken as the point at which 10% degradation occurs, T_0.1_, the mid-point of the degradation, T_0.5_, and the fraction of char which remains at the conclusion of the run [83].

For polymer/layered silicate nanocomposites, the incorporation of clay into the polymer matrix is generally found to enhance thermal stability by acting as a superior insulator and mass transport barrier to the volatile products generated during decomposition, as well as by assisting in the formation of char after thermal decomposition [49,84].

Chigwada [85] reported that thermal stability of polystyrene/OMMT nanocomposites is improved relative to that of pristine polystyrene. The T_0.1_ and T_0.5_ of nanocomposites with 3% OMMT loading increase by 14 °C and 16 °C, respectively.

The work of Liu *et al.* [86] reveals that thermal degradation of boron phenolic resin (BPR) and BPR / modified-MWCNTs nanocomposites takes place through a one-step process. The T_d_, defined as the temperature at 5% weight loss, of BPR is about 434 °C, while this temperature is shifted 36 °C to higher temperature when 1 wt % of MWCNT is added; it is clear that the addition of MWCNTs leads to a remarkable increase of BPR thermal stability. The stabilization effect of m-MWCNTs is mainly attributed to good matrix–nanotubes interaction, thermal conductivity of the nanotubes, as well as the barrier effect. Likewise, the char yield at 800 °C of the nanocomposites increases from 66% in BPR to 72% when 1 wt % of MWCNT is added [86].

### 3.2. Cone Calorimetry

The cone calorimeter is one of the most widely used methods for assessing the flammability of polymeric materials and is the most effective bench-scale method for studying fire retardancy. The cone calorimeter monitors a comprehensive set of fire properties in a well-defined fire scenario. These results can be used to evaluate materials’ specific properties, setting it apart from many of the established fire tests which are designed to monitor the fire response of a certain specimen. The cone calorimeter evaluates ignition followed by subsequent flaming combustion. The time to ignition depends on the thermal inertia, critical heat flux and critical mass loss for ignition, or alternatively the critical surface temperature for ignition. Fire response data obtained from cone calorimeter include mass loss, heat release rate, total heat release, smoke production, and CO and CO_2_ production, *etc.* Fire response properties more typical of fully developed or post flashover fire scenarios are not replicated in the cone calorimeter [87].

The heat release rate information from the cone-calorimeter is important to evaluate the flammability performance of polymeric materials [4,80]. Cone calorimetric analysis of different polymeric nanocomposites reveals significant improvements in flammability properties. The results of this analysis are expressed in terms of various combustion relevant properties, like heat release rate (HRR) and its maximum value (called peak HRR or PHRR), carbon monoxide yield, smoke release rate, *etc.*

The heat release rate is usually considered to be the most important piece of information that is obtained from the cone calorimeter. The calculation of the HRR is based on oxygen consumption principle, as described by Hugget [88]. According to this principle, for a given amount of oxygen consumed during the combustion process, the amount of heat released is constant and independent of type of the material undergoing combustion. The heat release rate, determined by oxygen consumption calorimetry, can be influenced by material specific properties, such as the specimen characteristics and the physical and chemical mechanisms active during the combustion. The PHRR is strongly dependent on the fire scenario as well as the intrinsic fire properties of the test specimen.

The total heat released (THR) during a cone calorimeter run is the integral of the HRR with respect to time- the total heat output up to that point - and is the fireload of the specimen in the cone calorimeter. For materials with a constant effective heat of combustion, the mass loss rate controls the HRR and the total mass loss controls the THR. Ignition occurs when the mass loss rate produces sufficient volatiles, at the characteristic air flow in the cone calorimeter, capable of ignition by a spark. Both CO production and smoke production result from incomplete combustion. Flame retardants working through flame inhibition result in a significant increase in the amount of CO and smoke yields in the forced flaming combustion of a cone calorimeter test [87]. CO production and smoke production play an important role that affect the safety of those trying to escape from the fire [89].

### 3.3. Limiting Oxygen Index (LOI) and UL-94 Protocol

The LOI test is one of the tests commonly used in the laboratory; it measures the oxygen content in an oxygen-nitrogen mixture that will sustain flaming combustion. The general assumption is that if more oxygen is required for combustion, a material will be more difficult to burn and thus fire retarded. In almost all cases, the oxygen index of polymer-clay nanocomposites is not increased and this is not an evaluation that is common for nanocomposites. The UL-94 protocol is designed to assess the ease of extinguishment of a plastic part; this is very dependent upon the sample thickness and thus this must be specified. As with the oxygen index, there are very few, if any, cases in which a nanocomposites can be classified by the UL protocol and thus it is not commonly reported for these systems.

## 4. Fire Retardancy of Polymer-Clay Nanocomposites

Nanocomposites have many outstanding properties, including fire retardancy, barrier effect and improved mechanical properties; probably the most important characteristic of these systems is that they provide all of these properties and thus they are multi-functional additives. One may choose to use a nanocomposite to enhance one particular property but one will obtain improvements in all of these, which is unusual for an additive.

**Figure 3:**
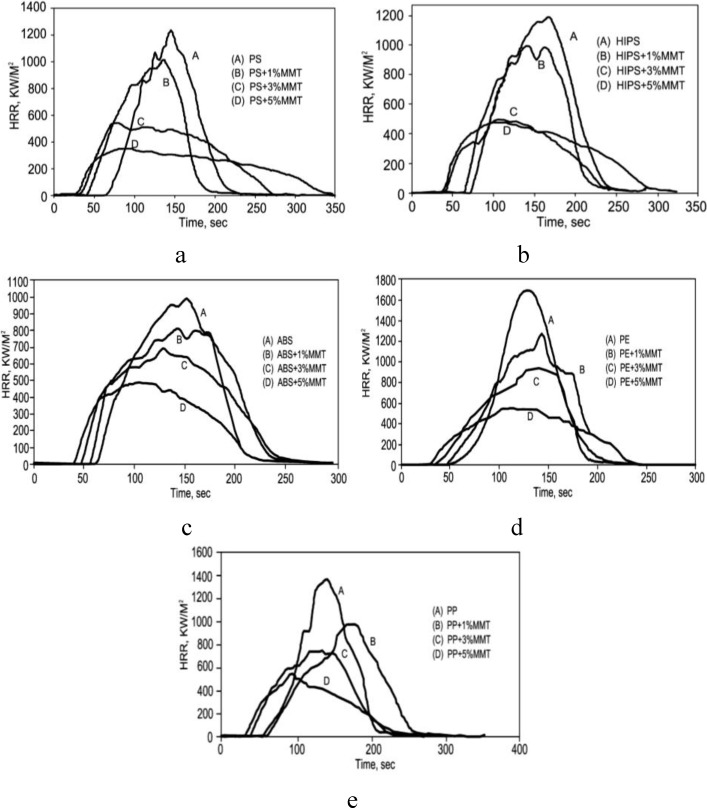
Heat release rate curves for (a) PS/MMT nanocomposites,(b)HIPS/MMT nanocomposites, (c)ABS/MMT nanocomposites, (d)PE/MMT nanocomposites and (e) PP/MMT nanocomposites. Reproduced with permission from reference [90].

Zheng *et al.* [90] reported the fire retardancy of PS/OMMT, HIPS/OMMT, ABS/OMMT, PE/OMMT, and PP/OMMT nanocomposites prepared by melt blending. Cone calorimetry indicated a substantial reduction in the peak heat release of all the nanocomposites, which mirrored a reduction in the mass loss rate, but there was an increase in amount of smoke evolved. The heat release rate curves for some of nanocomposites are shown in Figure 3; the reduction is very dependent on the amount of clay and 1% is, in general, not very effective. In some cases there is a larger reduction when 5% clay is used while in other cases, 3% and 5% clay are about equally effective. The relationship between the amount of clay and the reduction in PHRR is one of the questions that still must be answered for these systems.

A variety of polymer-MMT nanocomposites have been evaluated by cone calorimetry and it is found that the reduction in the PHRR is quite dependent upon the particular polymer. For instance, PMMA [91,92] gives the lowest reduction, about 25%, while PS [2], EVA [93] and PA-6 [94] are all at about 60% reduction. ABS and HIPS [95] fall in-between these extremes at about 40 to 45% reduction in the PHRR. In the discussion of the mechanism by which fire retardancy occurs, an explanation will be offered for these different values.

**Figure 4:**
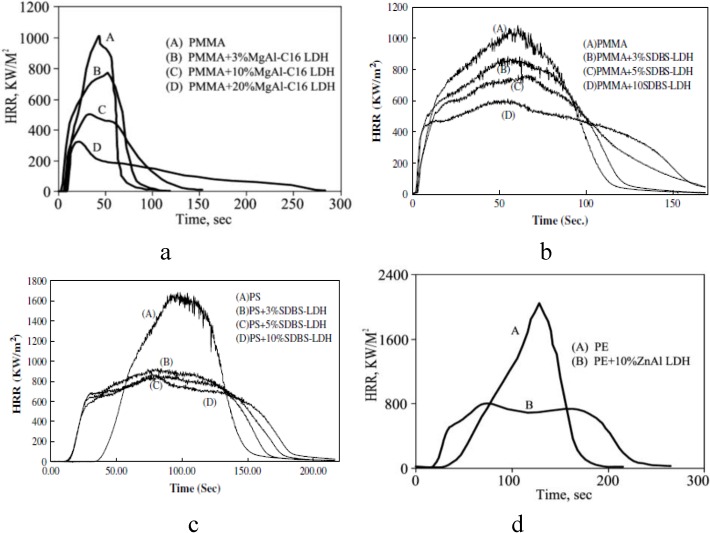
Heat release rate curves for (a, b)PMMA/LDH nanocomposites,(c)PS/LDH nanocomposites and (d) PE/LDH nanocomposite. Reproduced with permission from reference [14,64,107].

For polymer/LDHs nanocomposites, there are similarities, but also differences, with the behavior of polymer-MMT systems. In all cases with LDHs, the amount that is required is much larger than with an MMT; typically about 10% loading of an LDH is needed to achieve a reduction in the PHRR which is comparable to that obtained when 3% MMT is used (Figure 4). In some cases, Figure 4a, there is a rather large dependence on the amount of the LDH while in others, Figure 4c, changing the amount of the LDH has almost no effect. Polymer-LDH systems require further study to understand how and why they are effective.

Compared to LDH, it is relatively easy to obtain good dispersion of MMT in polymers. The reduction that is obtained for an LDH must follow a different mechanism than does MMT, since the LDH is rarely obtained with what one may call good nano-dispersion and thus the reduction in the PHRR with an LDH cannot be attributed to dispersion and it is usually ascribed to some combination of endothermic decomposition, formation of a glassy surface deposit with perhaps some contribution from the dispersion [83,96,97,98,99,100,101,102,103,104,105,106,107,108,109]. One goal for future work is to obtain a well-dispersed LDH in order to ascertain how much of the reduction in the PHRR is attributable to dispersion.

Rakhimkulov *et al.* [110] investigated the flammability of PP/MWCNT nanocomposites using the cone calorimeter. The maximum heat release rate for pristine PP is 2076 kW/m^2^, whereas those for the PP/MWCNT nanocomposites (at 1, 3 and 5 wt %), are 729, 553, and 456 kW/m^2^, respectively; the peak heat release rates decreased by 65, 73, and 78% [110].

## 5. Mechanisms by Which Polymer-MMT Nanocomposites Reduce the PHRR

In general, the popularly accepted mechanism to explain the reduction of peak heat release rate for polymer-MMT nanocomposites is based on barrier effects [111,112,113,114]. The combustion process of polymers is a complex coupling of energy feedback from a flame to the polymer surface with gasification of the polymer to generate combustible degradation products [115]. When the polymer matrix is heated to thermal degradation temperature, volatile flammable products are released from the polymer matrix, which combust after mixing with O_2_ at the matrix surface. The reduction of PHRR of polymer-montmorillonite nanocomposites is due to the accumulation of clay, perhaps at the surface or perhaps within the polymer. The accumulation at the surface will insulate the polymer from the heat and thus prevent degradation and it will also serve as a barrier to mass transport from the polymer to the vapor phase. Within the polymer, individual clay layers can inhibit the diffusion of radicals so that radical recombination reactions can occur, thus reforming new polymers which will again have to undergo degradation; this has been termed nanoconfinement [116]. This has the effect of spreading out the degradation over a longer time period.

The work of Kashiwagi and coworkers has shown that the formation of a network structure of nanoparticles with a polymer matrix can significantly reduce nanocomposite flammability. It is believed that one flame retardant approach is to suppress the bubbling rate - the superheated degradation products nucleate to form bubbles during combustion - so as to reduce the supply rate of fuel by forming a protective and heat shielding char layer. The formation of solid jammed network structure consisting of nanoparticles with tangled polymer chains can inhibit the vigorus bubbling process during combustion [22,77].

At a clay level of about 10% by mass, a network structure is formed for PS and the PMMA clay nanocomposites; it requires a level of about 0.5% with the SWNT and 2% with the MWNT. The tubes with their large aspect ratio, dense entanglement network and with strong bridging interaction form a physically stronger network compared to the less entangled clay platelet. This can explain why the flammability properties of clay-based polymer nanocomposites are not as good as those of carbon nanotube-based nanocomposites at relatively low particles concentration [77].

**Figure 5:**
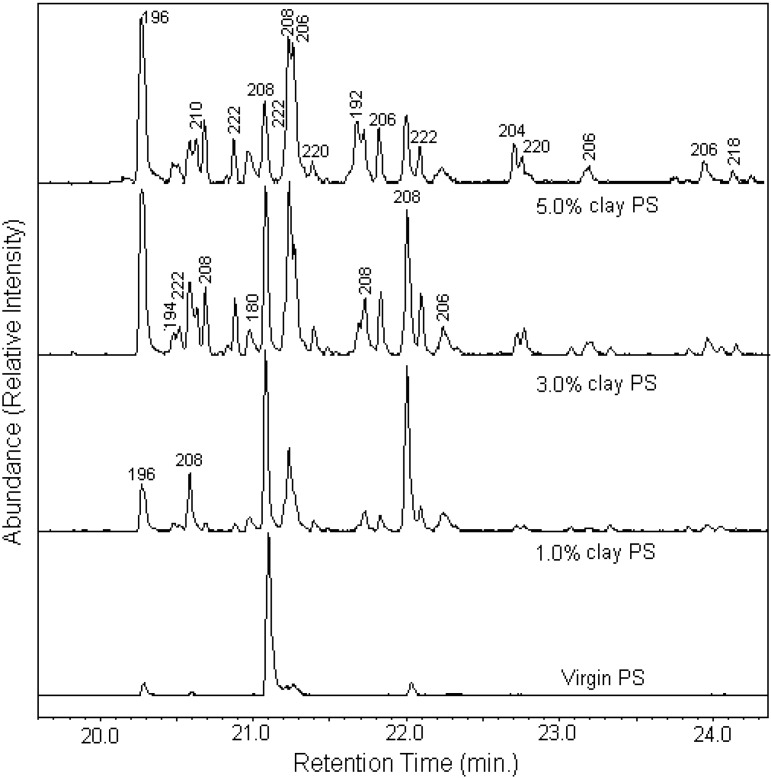
GC-MS traces for virgin PS and its nanocomposites containing varying amount of organically-modified clay. Reproduced with permission from reference [117].

In a systematic study on the effect of clays on thermal degradation and fire retardancy, it was found that most often the products of thermal degradation of a polymer-MMT nanocomposite are different from those of the virgin polymers [94,117,118,119]. Only one exception to this statement has been found and that is with PMMA. Here the degradation pathway is an unzippering reaction which is apparently not affected by the presence of the clay [120]. When a polymer-MMT nanocomposite degrades, it will form radicals which are, at least momentarily, nano-confined by the clay layers which are nearby and this momentary nanoconfinement permits radical recombination reactions which means that a new polymer is reformed which must again undergo degradation; this has the effect of spreading out the time for thermal degradation or combustion, exactly what is observed for the polymer-clay nanocomposites. The change in the composition of the degradation products for polystyrene nanocomposites only in the mass region where two benzene rings occur is shown in Figure 5. For virgin PS only a single peak, attributable to the styrene dimer, is seen while many peaks are seen for the nanocomposites and the amount of these new products increase as the amount of clay, and hence the amount of nanoconfinement, increases.

This has been extended to other nano-dimensional materials, notable LDHs and CNTs [96,120]. The results are a bit ambiguous – because there is a significant difference in the dispersion, one observes that the same products are obtained from the (nano) composites as are seen with the polymer. For the best dispersed systems, there may be a change in the product distribution but this is not certain. All that can be said with certainty at this time is that the process by which LDHs and CNTs provide a reduction in the PHRR is uncertain and this must be further investigated.

**Figure 6:**
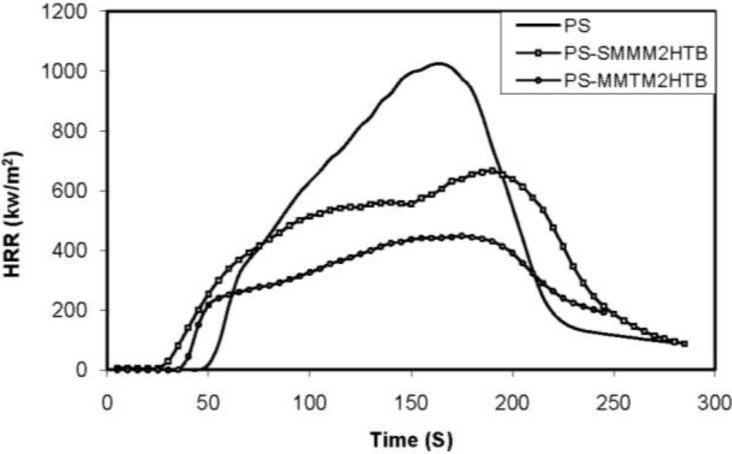
Heat release rate plots for polystyrene, and a polystyrene nanocomposite containing iron (MMT) and one in which iron is absent (SMM). Reproduced with permission from Reference [113].

The presence of paramagnetic iron naturally occurring in clays allows for radical recombination reactions with the clay, preventing degradation. The investigation of Zhu *et al.* indicates that clays which contain iron show enhanced thermal stability whether measured by TGA or cone calorimetry. It then appears that structural iron is the operative site for radical trapping within the clay. On the other hand, iron appears to have no role in the thermal stability of graphite-polystyrene nanocomposites, since it is not nano-dispersed as the structural iron is in the clay [113]. The heat release rate plots for PS nanocomposites with (MMT) and without (SMM) iron are shown in Figure 6. It is obvious that the plots are quite different when iron is absent and that it plays a role in the burning process.

## 6. Extension to Other Nanomaterials

Other nanomaterials are also used to prepare nanocomposites, e.g., graphite and graphite oxide, nanoscale silica particles, metals or metal oxides, metal hydroxides, cellulose whiskers, *etc.*

### 6.1. Graphite and Graphite Oxide

Graphite and graphite oxide nanocomposites have been prepared and evaluated in terms of fire retardancy for PS, HIPS, ABS, PA-6 and PMMA [112,121,122,123,124]. The reductions in the PHRR for graphite are quite comparable to those obtained using MMT as the nano-dimensional material. The reduction for PS is 48%, 36% for HIPS and 48% for ABS, 62% for PA-6 and 35% for PMMA. Thus it is likely that one can use graphite as a replacement for MMT. MMT does have an advantage in that the mechanical properties are enhanced while this does not happen with graphite. On the other hand, graphite nanocomposites should be able to conduct electricity and there may be situations in which this is important. Another obvious disadvantage for graphite nanocomposites is that they are black rather than transparent as are those of MMT. More work must be carried out with graphite as the nano-dimensional material to permit an evaluation of its relative merits.

**Figure 7:**
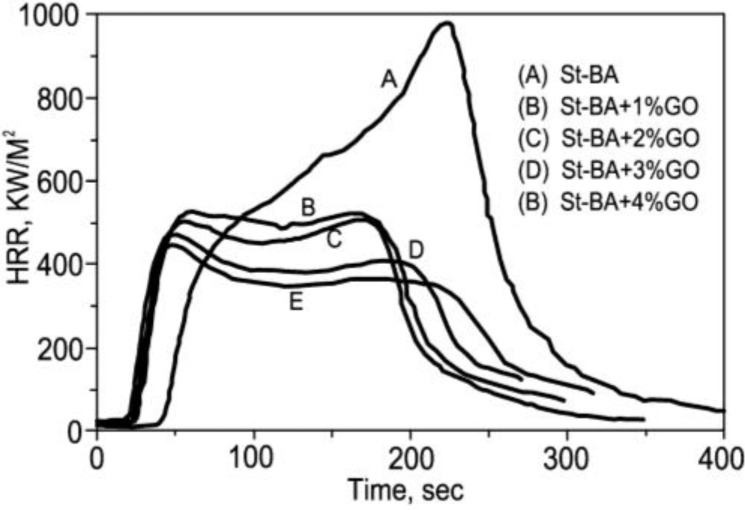
Heat release rate curves for styrene–butyl acrylate copolymer/graphite oxide nanocomposites with different graphite oxide mass fractions. Reproduced with permission from reference [125].

Zhang *et al.* [125] investigated the flammability and thermal stability studies of styrene-butyl acrylate copolymer/graphite oxide nanocomposite. The PHRR is reduced by 45% in an styrene-butyl acrylate copolymer / graphite oxide nanocomposite with graphite oxide content as low as 1 wt %. Furthermore, this system can also decrease the total smoke production and the smoke release rate during the combustion. Graphite oxide can be used as a flame retardant additive to obtain halogen-free, non-toxic, low-smoke and green flame retardant materials. This study will be beneficial to the further investigation and development of new ecological fire retardants. Figure 7 illustrates the heat release rate (HRR) for styrene–butyl acrylate copolymer /graphite oxide nanocomposites with different graphite oxide mass fractions.

### 6.2. Nanoscale Silica Particles

Nanoscale silica particles have a huge interfacial area because the diameter of the particles is in the nanometer range. Improved mechanical properties and thermal stability of polymer/ nanoscale silica nanocomposites has been reported [126]. The presence of nanosilica significantly reduced the heat release rate of the polymer matrix [127]. Ji *et al.* [128] reported the synthesis of poly(ethylene terephthalate) (PET)/SiO_2_ nanocomposites via the Sol–Gel method and the flame retardancy of the composites was improved. As compared to neat PET, PET/SiO_2_ nanocomposite containing 2.0 wt % SiO_2_ shows a lower HRR; this behavior can be explained considering that: 1) crosslinked structures are caused by SiO_2_ enhancing the interactions among macromolecules and 2) as an inorganic material, the SiO_2_ particles have inherent flame retardancy which can lead to a reduction in the combustion rate. In contrast to HRR, PET/SiO_2_ nanocomposites also show a significant decrease in the Total Heat Released as compared to neat PET. Therefore PET/SiO_2_ nanocomposites exhibit some improvement in fire retardancy compared to pristine PET [128]. The accumulation of silica on the surface of the burned polymer has been also observed in PMMA. In this study, two types of silica (fused silica and silica gel) were incorporated in two different molecular weight PMMA samples. The specific surface area of the silica and its porosity affected the thermal stability and flame retardant properties of the polymer by modifying the viscosity of the system in the molten state. Viscosity control proved to be a key-factor in the formation of the protective layer [127].

Kashiwagi *et al.* [129] prepared PMMA / colloidal silica nanocomposites by *in situ* radical polymerization of methyl methacrylate to study the effects of nanoscale silica particles on the mechanical and flammability properties of PMMA. The addition of nanosilica particles (13% by mass) did not significantly change the thermal stability, but it made a small improvement in modulus, and it reduced the peak heat release rate roughly 50%.

Yang *et al.* [130] investigated the flammability of polymer-silica nanocomposites. TGA results indicate that PMMA-silica nanocomposites show higher degradation temperatures than pristine PMMA; an increase in degradation temperature with increasing silica content and decreasing particle size is also found. Since there are more particles per weight for smaller size silica, more particles will offer more restriction sites for the polymer chain, and the scission of the polymer chain will become more difficult, and thus require more thermal energy for degradation. The better interfacial interaction between the additives and polymer chain introduced by the deeper penetration of smaller particles in the polymer matrix will also restrict the movement of the polymer chain. The LOI evaluation of PMMA–silica nanocomposites indicate that nanocomposites show very little improvement, although fillers in general lead to lower oxygen indices for thermoplastic samples due to decreased dipping.

### 6.3. Metal Oxides

The flammability of nanocomposites containing nanoscale titanium oxide, iron oxide and manganese oxides in polymer matrix has been investigated [131,132,133]. PMMA/TiO_2_ and PMMA/Fe_2_O_3_ nanocomposites were prepared by melt blending. The oxide is well distributed in the material but with some tendency to aggregation, the size of the aggregates being much less than 0.2 μm except for very few cases in which the size of the aggregates is in the micrometer range. The tendency to aggregation can be explained by the fact that no surface treatment was performed on the oxides. TGA analysis of PMMA/TiO_2_ and PMMA/Fe_2_O_3_ nanocomposites shown that the nanoparticles of TiO_2_ and Fe_2_O_3_ very significantly enhance the thermal stability of PMMA; this effect seems to be stronger in the case of TiO_2_ than for Fe_2_O_3_. For PMMA/TiO_2_ nanocomposites containing 20% wt TiO_2_, the PHRR decreased from 620 kW/m^2^ in pristine PMMA to 320 kW/m^2^. For PMMA/Fe_2_O_3_ nanocomposites containing 20% wt Fe_2_O_3_, the PHRR decreased from 620 kW/m^2^ in pristine PMMA to 400 kW/m^2^ [131].

Manganese oxide nanoparticles are also used for polymer nanocomposites. In an inert atmosphere, the thermal stability of the polypropylene is not affected by the addition of 10 wt % of MnO or Mn_2_O_3_. The situation is completely different in air; the degradation of the polypropylene filled with MnO and Mn_2_O_3_ starts 30 °C before that of pure polypropylene and the thermal stability is then significantly enhanced at higher temperatures. The temperature of maximum decomposition increases from 298 °C for pure PP to 371 and 379 °C for PP + 10 wt % Mn_2_O_3_ and PP + 10 wt % MnO, respectively. The heat release rates (HRRs) for knitted fabrics based on polypropylene filaments increase when polypropylene filaments filled with manganese oxide nanoparticles are used. The addition of fillers increases the PHRR and the time to ignition [132]. An increased time to ignition is advantageous while, of course, an increase in the PHRR is not desirable. It is difficult to understand how the addition of a non-burning oxide particle can increase PHRR.

### 6.4. Metal Hydroxides

The typical metal hydroxides used for polymer nanocomposites are magnesium hydroxide (MH) and aluminum hydroxide (alumina trihydrate ATH). Qiu *et al.* [134] used the surfactant-mediated solution method to synthesize nanoscale Mg(OH)_2_. Powders with the size of 3–6 nm in diameter and 50–100 nm in length for needle-like nanoparticles, or 3–10 nm in thickness and less than 100 nm in width for lamella-like nanoparticles were obtained. The value of LOI of the Mg(OH)_2_/EVA nanocomposite increases to 38 from 24 in traditional Mg(OH)_2_/EVA composites. The enhancement of flame-retardant property may be due to the good dispersion of Mg(OH)_2_ nanoparticles in EVA matrix and the formulation of the compact chars.

Ethylene vinyl acetate copolymer (EVA)/aluminum hydroxide nanocomposites were prepared by melt-blending. Pure EVA resin is flammable with an LOI of 17, and ATH exhibits flame retardancy at high loadings. For instance, the addition of 60 wt % of untreated ATH gives an LOI of 30 and a V-2 rating in the UL-94 protocol. In comparison to the previous case, the flame retardancy of the coupling agent treated system is improved, *i.e.*, this specimen obtained a V-1 UL-94 classification with an LOI of 37 [135].

## 7. Combinations of Nanomaterials with Conventional Fire Retardants

At one time, it was thought that the addition of the organically-modified clay would solve the problem of fire retardancy. It is now well known that this is not true and that, in fact, all we can do is to reduce the peak heat release rate but we do not affect the ignitability or extinguishability. Therefore the clay can be a part of the FR solution for some polymers but it is only one part. Nanomaterials have been used together with conventional fire retardants in the polymer matrix to see if they can interact to develop a system which is more effective looking at all aspects.

The combination of aluminum hydroxide with the organically-modified clay has been studied in EVA and compared with the classic (ATH only) system. Traditionally one uses 65% ATH and 35% EVA in a wire and cable situation and this gives a PHRR of about 200 kW/m^2^ at a heat flux of 50 kW/m^2^. When 5% of the ATH was replaced by the clay, the PHRR dropped to 100 kW/m^2^. With EVA – ATH only, one needs 78%ATH to obtain this value. If 200 kW/m^2^ is sufficiently low, then one can decrease the ATH from 65% to 45% and add 5% clay and thus 50% EVA. This leads to not only improved fire performance but also to improved mechanical and rheological properties [136].

A similar investigation has been carried out on polypropylene. A 76% reduction in PHRR is seen when 60% PP and 40% ATH are combined. An equivalent reduction is observed with only 20% ATH by the addition of 5% organoclay, thus increasing the amount of polymer very substantially [137,138].

If one adds triphenylphosphate (TPP) to polystyrene, there is severe plasticization of the polymer so that it will actually flow. When the clay is present, the plasticization is reduced and some measure of fire retardancy is achieved. The rather typical situation for a nanocomposite is that the peak heat release rate is lowered but the total heat released is unchanged, which means that eventually everything does burn but it takes a little longer time. When 30% of the phosphate is present, there almost an 80% reduction in PHRR with almost a 60% reduction in the total heat released [139].

Copolymers of styrene and dibromostyrene were used to make nanocomposites. A polymer which contained only 10% dibromostyrene and 3% organically-modified clay gave a reduction in the peak heat release rate of 72% and a V-2 classification in the UL-94 protocol [140].

The reaction to fire of polymer nanocomposites (thermoplastic polyurethane and polyamide-6) containing two different nanofillers (organoclay and carbon nanotubes) has been investigated. Polymer nanocomposites exhibit significant reduction of peak of heat release rate but the nanomorphology (exfoliation, intercalation and presence of tactoids) does not play any significant role, although a reasonable level of nanodispersion is necessary to achieve good flame retardancy in specific cases (mass loss calorimetry experiment). Modeling for the time to ignition is also proposed. It is shown that the nanocomposite approach gives the best results combined with conventional flame retardants (phosphinate and phosphate) and leads to synergistic effects [141].

The recent work on PP/LDH (nano)composites shows that there are no significant change in PHRR reductions for the PP/organo-LDHs (nano)composites, the PHRR reductions of PP/organo-LDHs (nano)composites were 0, 6 and 30% at 3, 5 and 10% LDHs loading, respectively. On the other hand, when 10% of zinc borate was addded to the PP composites, the PHRR reductions changed significantly. The PHRR reductions of PP/10% BZn was 38%, and the reductions of PP/organo-LDHs/10%BZn (nano)composites at 3, 5 and 10% organo-LDH loading are 48, 50 and 63%. This implies, but does not prove, synergy between the organo-LDHs and zinc borate for PP.

## 8. The Future of Nano-Dimensional Materials in Fire Retardancy

We expect that in the future nano-dimensional materials will be a part of commercial fire retardant additives. In the past, fire retardants have usually been a single compound (perhaps in combination with a synergist as with Sb_2_O_3_ and bromine compounds). We think that nano-dimensional materials will play a role; the enhanced mechanical properties that arise when MMT is used, for instance, may be of value to offset the reduction in mechanical properties due to some additives.

In order to aid in the development of these products, researchers must identify the processes by which nano-dimensional materials can effectively reduce the PHRR so that one can combine mechanisms to achieve fire retardancy. In the case of LDHs, one must determine how to obtain an LDH well-dispersed in a polymer to see if dispersion plays any role in the fire retardancy with these materials. Far and away the most commonly investigated nano-dimensional material is MMT and work must continue with this material to refine its use and identify the optimal loading at which it should be used. Carbon nanotubes are currently very popular and are being more and more investigated as the price of this material falls. This will continue and CNTs may well become more important; the single disadvantage is the color. New growth will occur with other nano-dimensional materials which are not now under serious investigation for fire retardancy. It is important to investigate these novel materials, such as metal oxides, sulfides and phosphates, to see if they can be well-dispersed in polymers and how they affect the properties of those polymers.

## Acronyms

ABS: Acrylonitrile-butadiene-styrene terpolymer
AFM: Atomic force microscopy
ATH: Alumina trihydrate
BPR: Boron phenolic resin
BZn: Zinc borate
EVA: Poly(ethylene-co-vinyl acetate)
GC-MS: Gas chromatograph-mass spectrometer
HIPS: High impact polystyrene
HRR/RHR: Heat release rate
LDH: Layered double hydroxide
LOI: Limiting oxygen index
MH: Magnesium hydroxide
MMT: Montmorillonite
MWNT/MWCNT: Multi-wall carbon nanotubes
m-MWCNT: Modified multi-wall carbon nanotubes
NMR: Nuclear magnetic resonance
OMMT: Organically modified montmorillonite
PA-6: Polyamide-6
PE: Polyethylene
PET: Poly(ethylene terephthalate)
PHRR: Peak heat release rate
PMMA: Poly(methyl methacrylate)
PP: Polypropylene
PS: Polystyrene
SEM: Scanning electron microscopy
SWNT/SWCNT: Single-wall carbon nanotubes
TEM: Transmission electron microscopy
TGA: Thermogravimetric analysis
THR: Total heat released
TPP: Triphenylphosphate
UL-94: Underwriter’ Laboratory Test #94
XRD: X-ray diffraction
